# Atmospheric Purification

**Published:** 1886-07

**Authors:** 


ATMOSPHERIC PURIFICATION.—By David Prince, M. D. This treatise in the form of a pamphlet of some 20 pages, is written with the conviction that it presents an advance in the art of avoiding some of the enemies of life.

